# Supplementary material for: Prestige bias drives the viral spread of content reposted by influencers in online communities
Source: Sci Rep. 2025 May 1;15:15282. doi: 10.1038/s41598-025-98955-4 (PMC12046028; doi:10.1038/s41598-025-98955-4)
Supplement: Supplementary file 1 — Supplementary Information. [file 41598_2025_98955_MOESM1_ESM.pdf]

Supplementary Materials: Prestige bias drives the viral spread of content reposted by influencers in online communities

Takuro Niitsuma*[1], Mitsuo Yoshida[2], Hideaki Tamori[1], and Yo Nakawake*[3]

[1]Media R&D Center, The Asahi Shimbun Company, 5-3-2 Tsukiji, Chuo-ku, Tokyo, Japan
[2]Institute of Business Sciences, University of Tsukuba, 3-29-1 Otsuka, Bunkyo-ku, Tokyo, Japan
[3]Graduate School of Advanced Science and Technology, Japan Advanced Institute of Science and Technology, 1-1 Asahidai, Nomi, Ishikawa, 923-1211, Japan

S1 Detail of English Dataset

In addition to our main dataset analyzed in this study, we also examined an English-language dataset to check the robustness of our findings. Specifically, we used a set of one million users (sampled in December 2014 from the global timeline) provided by Yamaguchi et al. [3] and collected all reposts over 24 hours on January 1, 2015. Note that because these users were sampled from the global timeline, they may not all be English speakers.

As in our main analysis, we applied the same hg index-based approach to measure user influence. However, because this dataset covers only a single day, the resulting hg index values are less fine-grained than our main dataset, which spans 31 days of Japanese posts. Consequently, we classified users into four categories (low, mid, high, and very high) rather than six.

Furthermore, owing to the limited temporal coverage (24 hours), we analyzed the cascading repost probability (CRP) in shorter time windows (30 minutes, 1 hour, and 3 hours). Although the observation period is more constrained than our main analysis, we observe similar trends: users in the very high influence group consistently achieve higher CRP. We also note that some typically influential users may be underrepresented or misclassified due to the brief window. Therefore, we should interpret these results as a supplemental check rather than a comprehensive analysis of long-term user behavior and diffusion dynamics.

S2 Topics of User Profiles

User profiles used for the regression analysis were tokenized using the Japanese tokenizer Mecab [2], splitting the text into word-level tokens. As preprocessing, we removed stop words, function words, URLs, and mentions. After this, we applied **Biterm Topic Modeling (BTM)**[4], which is particularly well suited for short texts. For simplicity, we set the number of topics to 10. Table S1 provides an overview of these 10 topics, including example Japanese keywords and their approximate English translations for reference.

Table S1: Overview of 10 Topics of User's Profiles with Example Keywords (Japanese). English keywords in parentheses are provided for reference.

Topic	Label	Example Keywords (JP / EN)
1	Fandoms of Male Artists	ハート(heart), 応援(support), ファン(fan), SMAP (artist name), 嵐(Arashi / artist name)
2	Raffles and Others	懸賞(raffle), 当選(winner), 旅行(travel), 猫(cat), コスメ(cosmetics)
3	English Phrases	I, AND, of, TO, IS
4	Gaming and Anime Fandoms	ウマ娘(Uma Musume / game title), FGO (game title), アイコン(icon), 描く(draw), ポケモン(Pokemon / game title)
5	Political Views and Opinions	日本(Japan), 反対(oppose), 政治(politics), 自分(self), コロナ(COVID-19)
6	Fandoms For Female Content	腐る(decay), FGO (Game) , 夢(dream), ツイステッドワンダーランド(Twisted Wonderland / game title), 腐女子(fujoshi)
7	Bussines Use	DM (direct mail), 情報(information), 依頼(request), お仕事(job), イベント(event)
8	Sports and Entertainment Fandoms	野球(baseball), サッカー(soccer), 音楽(music), ライブ(live), 乃木坂46 (Nogizaka46 / artist name)
9	Drawing Community	描く(draw), 絵(picture), アイコン(icon), ツイート(tweet), イラスト(illustration)
10	Miscellaneous	趣味(hobby), 音楽(music), 映画(movie), 写真(photography), 漫画(manga)

S3 Topic Analysis on Random Effects

This section describes the random effects of the mixed-effects model in subsection 2.4, mainly focusing on posts. Additionally, we provide an analysis of the topics of these posts.

In our main analysis, we fitted a mixed-effects model using the `lme4`[1] package in R, specifying random intercepts for both `source_tweet_id` and `user_topic`. Table S2 summarizes the random effects that Table 2 in the main analysis. The standard deviation for `source_tweet_id` (0.7733) is higher than that for `user_topic` (0.4321). This indicates that each tweet captured by `source_tweet_id` contributes more to whether a post reposts than differences among user topics. Notably, the standard deviation of `source_tweet_id` is larger than the coefficients of `sender_influence` for *very high* users.

Table S2: Random effects from the mixed-effects model.

**Groups**	**Name**	**Variance**	**Std.Dev.**
`source_tweet_id`	(Intercept)	0.5980	0.7733
`user_topic`	(Intercept)	0.1868	0.4321

To explore whether topical differences might explain the sizeable post-level variation, we applied the same model (BTM) for the topics of user profiles to the dataset of source tweets. We tested 10, 15, and 30 topics, and the 10-topic model yielded the highest coherence. Table S3 provides an overview of these 10 topics, including example Japanese keywords and approximate English translations for reference.

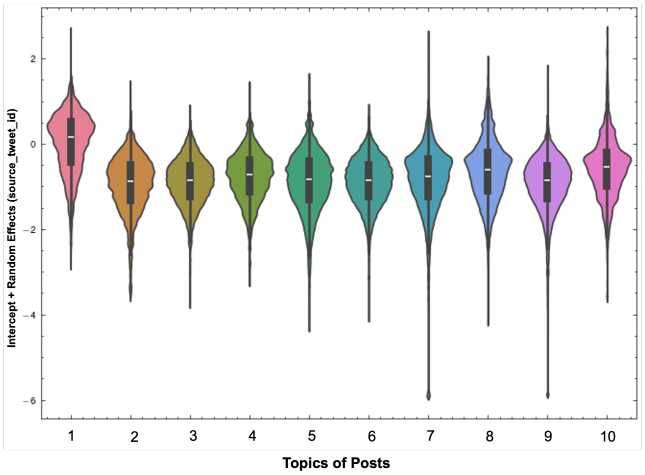

Figure S1: Sample visualization of intercept + random effects for each topic.

Table S3: Overview of 10 Topics of Posts with Example Keywords (Japanese). English keywords in parentheses are provided for reference.

Topic	Label	Example Keywords (JP / EN)
1	Promotional Campaigns & Giveaways	プレゼント(present), フォロー(follow), キャンペーン(campaign)
2	Miscellaneous Daily Life	猫(cat), 犬(dog), 食べる(eat), 地震(earthquake), 水(water)
3	Political / Election-Related	選挙(election), 投票(vote), 政党(political party), 政権交代(change of government)
4	Sports Events (Baseball, Soccer, etc.)	選手(player), 試合(match), 優勝(victory), チーム(team)
5	Creative Works, Illustrations, Gaming	イラスト(illustration), ゲーム(game), デザイン(design), 描く(draw)
6	COVID-19 and Government Policy	ワクチン(vaccine), 接種(inoculation), コロナ(COVID-19), 政府(government)
7	Positive Emotions and Festive Themes	笑顔(smile), ハロウィン(Halloween), 嬉しい(happy), 誕生日(birthday)
8	Media, Broadcasting, and Streaming	配信(streaming), 放送(broadcast), 映画(movie), 動画(video)
9	General Opinions and Everyday Reflections	思う(think), 言う(say), 自分(myself), 見る(see)
10	Event Announcements and Ticketing	開催(holding an event), チケット(ticket), 予約(reservation), 会場(venue)

Figure S1 shows the model's intercept plus its random effect for each `source_tweet_id`, illustrating whether these distributions differ substantially by topic. In short, no topic stands out as having an especially high or low average repost likelihood. This suggests that, while the unique content of each post strongly influences its chances of being reposted, it is not simply a matter of belonging to one of the 10 broad topics. Consequently, factors such as emotional valence, writing style, timing, or visual elements may play larger roles in driving repost behavior. These considerations remain open areas for future investigation.

S4 Analysis of Influencers' Reposting Behavior

To further investigate the cause of influencers' high share in the secondary spread, we analyzed users' reposting behavior by aggregating the number of reposts for each user and visualizing the proportion of reposts accounted for by top-reposted users (Figure S2).

Figure S2(a) reveals that the top 1% of reposted users account for 30% of all reposts, whereas the top 20% account for 80%. Importantly, these top-reposted users comprise users with various levels of influence. The Complementary Cumulative Distribution Function (CCDF) in Figure S2(b) demonstrates that while the majority of users make very few reposts, a small number of users make a disproportionately large number of reposts.

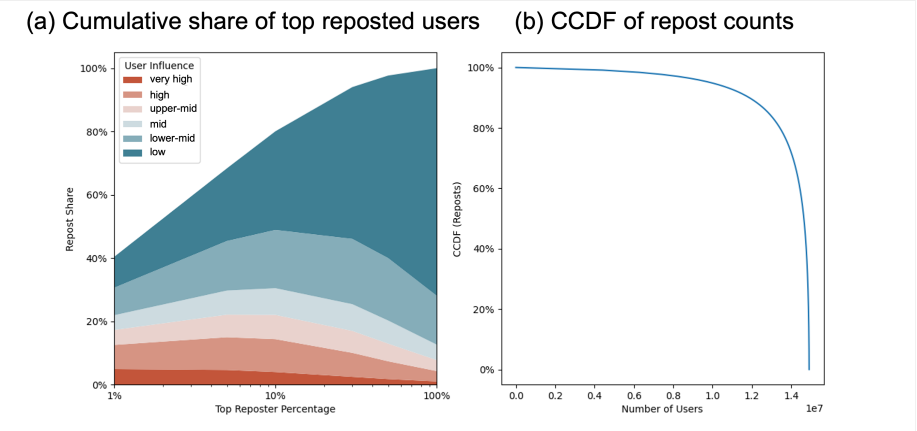

Figure S2: Analysis of users' reposting behavior. (a) Cumulative share of top reposted users. (b) CCDF of repost counts.

These findings suggest that the high share of influencers in the secondary spread is not merely due to their higher frequency of reposting. This result indicates that the influence category significantly affects the information distribution efficiency in the secondary spread. Information reposted by influencers tends to reach a wider audience and is more likely to be reposted than information shared by other user groups, which is consistent with prestige bias.

Additionally, the results show a substantial skew in the number of reposts among users. Therefore, a little of users spread the most of reposts, indicating potential bottlenecks in information diffusion and the risks of excessively spreading certain information. These results and our main results demonstrate that influencers' high share in the secondary spread is attributed to the qualitative aspects of their influence rather than simply a higher volume of reposts. This analysis highlights the issue of skewed information diffusion as a new area for future research.

S5 Sensitivity Analysis Within 30 Minutes of Posting

Although our primary analyses used a virtual timeline constructed in a near-chronological manner, we acknowledge that X (formerly Twitter) provides algorithmic recommendations such as trending topics, which can reorder or highlight posts in ways that deviate from strict chronological sequence. To reduce these algorithmic effects, we limit our analysis to the first 30 minutes after a post's publication. Trending or recommended content typically relies on accumulating engagement signals over a longer timeframe.

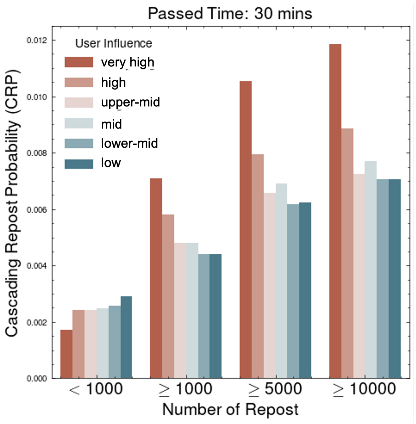

Figure S3: Cascading Repost Probability (CRP) within 30 minutes of the original post, broken down by user influence categories and post popularity thresholds.

Figure S3 shows that even in this shorter timeframe—where the influence of algorithmic ordering may be relatively limited—the observed trends in repost behavior remain consistent with our main findings. Specifically, higher influence users continue to exhibit relatively higher CRP values for popular posts (e.g., $\geq$ 1000 reposts), suggesting that the prestige bias effect is robust under these near-chronological conditions.

We note, however, that some algorithmic prioritization could still occur within these initial 30 minutes. Nevertheless, the consistency of these results supports the view that our near-chronological virtual timeline reasonably approximates user exposure patterns, capturing meaningful insights about information diffusion and user influence in the early stages of reposting.

References

[1] Douglas Bates, Martin Mächler, Ben Bolker, and Steve Walker. Fitting linear mixed-effects models usinglme4. *Journal of Statistical Software*, 67 (1), 2015. ISSN 1548-7660. doi: 10.18637/jss.v067.i01. URL `http://dx.doi.org/10.18637/jss.v067.i01`.

[2] Taku Kudo. Mecab : Yet another part-of-speech and morphological analyzer. *http://mecab.sourceforge.net/*, 2005. URL `https://cir.nii.ac.jp/crid/1572543025344508032`.

[3] Yuto Yamaguchi, Mitsuo Yoshida, Christos Faloutsos, and Hiroyuki Kitagawa. Patterns in interactive tagging networks. *Proceedings of the International AAAI Conference on Web and Social Media*, 9(1):513–522, August 2015. ISSN 2162-3449. doi: 10.1609/icwsm.v9i1.14616. URL `http://dx.doi.org/10.1609/icwsm.v9i1.14616`.

[4] Xiaohui Yan, Jiafeng Guo, Yanyan Lan, and Xueqi Cheng. A biterm topic model for short texts. In *Proceedings of the 22nd international conference on World Wide Web*, WWW '13, page 1445–1456. ACM, May 2013. doi: 10.1145/2488388.2488514. URL `http://dx.doi.org/10.1145/2488388.2488514`.
